# Investigating social media addiction and impact of social media addiction, loneliness, depression, life satisfaction and problem-solving skills on academic self-efficacy and academic success among university students

**DOI:** 10.3389/fpubh.2024.1359691

**Published:** 2024-07-08

**Authors:** Imran Aslan, Hatice Polat

**Affiliations:** ^1^Faculty of Health Sciences, Health Management Department, Bingöl University, Bingöl, Türkiye; ^2^Faculty of Health Sciences, Department of Gerontology, Malatya Turgut Özal University, Malatya, Türkiye

**Keywords:** academic self-efficacy, social media addiction, loneliness, depression, life satisfaction, problem solving skills

## Abstract

**Introduction:**

The negative effects of post-COVID-19 restrictions have been detected in students’ mental well-being due to internet addiction, changing habits, despair and uncertainty. Students’ academic success is expected to be affected by social media addiction, loneliness, depression, life satisfaction, problem solving skills and academic self-efficacy factors. This study aimed to determine the level of social media addiction and the effects of these factors on the academic success of university students and define their interactions with each other.

**Methods:**

Four hundred nineteen questionnaires were collected between October–December 2022 at Bingöl University, Türkiye. Descriptive statistics, independent t-test, One-Way ANOVA, correlation and multiple linear regression methods were used to analyze data with the help of the SPSS 22 software.

**Results:**

Middle level grade (GPA) average (71,17 ± 9,69 out of 100), low level social support from friends and family members (34,6%), spending more than 4 h on social media (42,5%), middle level social media addiction, moderate depression level (51,31%-PHQ > 10), mild loneliness and slight dissatisfaction with life were found among students. Furthermore, high academic self-efficacy, moderate agreement with academic performance and good problem-solving skills were indicated in the survey results. Significant differences, such as higher life satisfaction among males and higher depression among females, were measured. Academic self-efficacy scale, problem solving skills and satisfaction with life had a negative correlation with social media addiction and depression, while a positive correlation with academic performance measures. Problem solving skills, satisfaction with life, fourth class vs. others and living alone vs. others were positive predictors of the academic self-efficacy. Meanwhile, loneliness was a negative predictor of the academic self-efficacy, while higher problem-solving skills and being female were positive factors leading to a higher GPA.

**Discussion:**

The fact that the participants were only students from Bingöl University limits the ability to generalize the results. Policymakers could implement social and problem-solving skills training to develop better academic programs and cognitive-behavioral therapy for students’ academic success.

## 1 Introduction

Students are more vulnerable to mental disorders and it is believed that the number of students addicted to social media had increased during the COVID-19 Pandemic due to movement restrictions and uncertainty, which had led to post-pandemic effects on students’ well-being and performance (1–3). Spending more than 3 h on social media platforms can lead to social media addiction, culminating in low mental health that affects work and academic productivity. Moreover, excessive social media usage can develop sleep loss problems, anxiety and depression (4). One study in Türkiye found higher levels of social anxiety and depression as well as lower self-esteem among students with internet addiction (5). However, social media can also decrease loneliness and depression (6).

Social media applications offer individuals the opportunity to facilitate communication and access information by removing various types of limitations. The young generation’s preoccupation with social media has rendered them incapable of resisting social media as an alternative to manage anxiety and stress. This situation causes some problems along with the positive features of social media, one of these problems being social media addiction. Excessive use and lack of control are main reasons for social media addiction or behavioral addiction disorder, which leads to social overload, envy and anxiety, similar to compulsive buying behaviors. Mood alternations, negative outcomes, and excessive time spent on social media result in loss of productivity and feelings of isolation that develop over time with psychological dependence on social media, which leads to users trying to overcome undesirable moods using social media (3, 7, 8).

Long-term psychological problems occur as a result of extreme stress and depression. Individuals who are alone tend to use social media more. Excessive use of social media can isolate people (3). Social media first affects individuals, and these individuals with psychological and behavioral changes then cause changes in the sociological, psychological and cultural characteristics of the society (9, 10).

Level of academic self-efficacy can influence the academic productivity and mental wellbeing of students. Problem solving skills can help students to define and determine why an issue is evolving and implement appropriate solutions that affect academic success and mental well-being during crisis situations, like the COVID-19 Pandemic, earthquakes etc. An individual’s differences in depression and social media addiction can be revealed and students’ academic success can be improved by examining relationships among related variables. This suggests that other circumstances should be taken into account when explaining social media addiction and depression since problem-solving skills and loneliness also have significant effects on students’ learning process and mental health problems. Moreover, minor mental problems can be detected in students with high life satisfaction. In the literature, this type of study has mostly used statistical methods such as descriptive, correlation and regression methods.

This study intended to determine the relationship between social media addiction, life satisfaction, depression, loneliness, problem-solving skills and academic self-efficacy, while also determining the predictors of academic self-efficacy and academic success based on the GPA and perceived self-success evaluation among university students in Türkiye. The study tried to combine their academic success based on cause-effect with self-evaluated and real academic success parameters. The main contribution of this study is to determine changes in social media addiction, depression, life satisfaction and their effects by analyzing loneliness, problem solving skills and academic self-efficacy in order to predict the academic performance of university students in Türkiye.

The study aimed to answer three main research questions: Firstly, what are the links between social media addiction, loneliness, depression, satisfaction with life of satisfaction, ability to solve problems, self-efficacy, and academic success in college students? Secondly, what are the changes in social media addiction pre, during, and after the COVID-19 periods? Finally, what the predictors of academic success and academic self-efficacy? This study hypothesized that there is a strong negative correlation and relationship between social media addiction, depression and loneliness with academic success and academic self-efficacy. In addition, there is a strong positive correlation and relationship between problem-solving skills, life satisfaction and academic success and academic self-efficacy. The research paper is organized as follows: Literature section (section 2) provides insight into academic self-efficacy, adult problem-solving skills, social media addiction, life satisfaction, depression and loneliness factors. Materials and methods (section 3) presents the study design and setting, participants and procedures, scales, and statistical methods used to analyze the data. In the next sections, the results (section 4) are presented and discussed, along with the study’s limitations and future recommendations (section 5). Finally, a brief conclusion is given in section 6.

## 2 Literature review

Academic self-efficacy and problem-solving skills are important capabilities required for managing undesirable situations and improving students’ academic performance. A student’s belief in successfully completing an academic task as an individual is a crucial part of academic success in-relation to self-efficacy. Loneliness and depression are related to social media addiction (4, 7), while loneliness alone is correlated to internet addiction, and severe symptoms of depression (11). Using the internet for more than 5 h was significantly associated with internet addiction and depression (12). Students with high levels self-efficacy are more willing to participate in academic activities and can develop effective strategies when in difficulties (7).

### 2.1 Academic self-efficacy

Academic success refers to the performance in education courses and transfer of knowledge. Students whose academic achievements increase tend to transition into their professional lives more competently and successfully (13). Academic self-efficacy refers to an individual’s belief in the ability to complete an academic task rather than the belief in personal attitudes and abilities. Academic self-efficacy helps students become more socially, emotionally and academically optimistic individuals. In addition, students with this trait are less likely to engage in risky behavior and can easily cope with difficult situations. In recent years, researchers have found that there is a strong link between academic self-efficacy and academic achievement. Studies show that a positive academic self-efficacy supports academic success (14, 15).

### 2.2 Problem solving skills for adults

A problem is an undesirable, distressing or complicated situation, such as an economic, emotional, or physical difficulty, leading to a disturbed individual. Obstacles and conflicts can prevent an individual from reaching one’s goals. Novel problems such as new jobs as well as novel tasks and goals can be managed with effective problem-solving skills in an uncertain and complex society and positive results can be obtained with problem-solving mechanisms, and choosing an effective solution among possible or alternative solutions (16). Making logical decisions and carrying out activities should be in accordance with goals and objectives. Problem-solving skills depend on an individual’s experience and rational problem-solving skills can be effective as a positive orientation toward problem solving (16, 17). Trial-and-error and internal or causal approaches are applied to find solutions to problems. Planning, implementing and evaluating actions using the social network and exchanging knowledge and information are some of the ways to solve problems (16, 18).

Analytic, flexible, specific, logical, structural, realistic and empathetic thinking and openness to new relationships are accepted as problem-solving competencies. Problem definition, generating alternative solution, decision-making, and implementing solutions are domains of problem-solving skills. Using tools interactively, interacting in heterogeneous groups, and acting autonomously are three main competencies required for adapting to a changing environment. Moreover, cognitive competencies (critical thinking and problem solving), intrapersonal competencies (flexibility and adaptability) and interpersonal competencies (communication and collaboration) are necessary for achieving high academic success. Positive emotions, such as joy or hope, are also aspects of problem-solving that help achieve goals. An individual with a positive problem-orientation approach feels confident in dealing with problems and challenges, called constructive problem-solving, better than impulsivity-careless style and avoiding problems style, categorized as a negative problem-orientation (18).

### 2.3 Social media addiction

Social media addiction is defined as a form of addiction that harms the social, physical and psychological functionality of a person through excessive and increased frequency of social media use over time, and the inability to limit internet use in spite it causing social, academic and mental problems (psychological dependency) that affects the daily activities in a person’s life. Escaping from stress and life problems as a way to continue interpersonal relationships can be reasons for using social media besides keeping in touch with friends, chatting, sharing interesting things, gathering useful information, disseminating information, gaining more contacts, making groups, selling or buying products. Inefficient self-regulation, neglect of personal life, cognitive preoccupation, mood modifying experiences, lack of tolerance, concealment of addictive behaviors, and escapism are signs of social media addiction (3, 7, 8, 19). Some of the negative impacts of excessive usage of social media on a user’s life (internet addiction) are salience, excessive thinking or planning to use social media, excessive time spent on social media, mood modification, social media as a tool for overcoming emotional problem, relapse or failure in decreasing social media usage, withdrawn or feeling troubled when unable to use social media and conflicts (20).

Studies have shown that people between the ages of 18–29 use social media more than older people (21). Situations such as not being able to quit or control a substance or behavior seen in addictions are also valid for social media addiction. Increase in the time spent on social media causes individuals to constantly update themselves, and at the same time, remain connected to social media in order to follow developments in their environment (22). Having easy internet access and increased usage time cause users to spend longer periods of time on the internet, without being aware that the time could be spent on finishing tasks with good intentions and determination. Social relationship problems, such as lacking close family relationships, can cause risk-taking behavior and individuals might turn to long-term internet use in efforts to seek new relationships, preferring communication via the internet instead of face-to-face communication that might be distressful and irritable, thus, spending even more time online (23).

Occasional users who spend less time on social media have lower levels of depression, anxiety and stress. Low risk users are high on tolerance and salience criteria. At-risk users are more prone to depression, anxiety, and stress, while problematic users have the highest level of problematic social media use, depression, anxiety, and stress (20). Social networks can cause problems such as decreased critical thinking abilities as part of problem solving-skills, attention deficit, hyperactivity disorder, lack in time management, decrease in the time allocated to reading and decline in school grades (24). Addicted young adults are more likely to show depressive symptoms (25). In addition, long-term use of social media leads to changes in people’s mood and personality patterns and their learning processes and academic life are influenced, especially during a crisis, as seen during the COVID-19 Pandemic (3).

### 2.4 Satisfaction with life

Satisfaction is one of the most important indicators of successful adaptation to life and it reflects a cognitively self-judging well-being relating to human life, work, family, physical and mental health etc. This trait is beneficial for health, longevity, and social relationships. Life satisfaction, income, job satisfaction, needs satisfaction, resilience, as well as social relationships and support are positive influencing factors of satisfaction, while unemployment, stress, anxiety, and depression are negative factors (26). A lower risk of depression in students is associated with a satisfied life. People who are more satisfied with their lives are expected to have a positive mood and successful academic activities (1, 2). Students with low life satisfaction during COVID-19 were found to be affected by the lockdowns, economic problems, fear of infection, social media influence etc (27).

### 2.5 Depression

Depression has increased recently around the world, including Türkiye, due to the COVID-19 Pandemic, earthquakes and the worsening economic situation. This is evident in depressive mood swings, despair, loss of interest, loss of energy, feelings of worthlessness etc. Students are inclined to feel depressed due to low academic achievements, and a lack of employment and earning opportunities in the future. A lack of social interactions can also lead to depression (1, 28). Females and last-term students have shown a higher prevalence of depression due to worries of not securing a job after graduation (27, 29). Suicide rates have increased in tandem with increased depression rates among young students, whereby one in five students have reported suicidal ideation (30).

### 2.6 Loneliness

A discrepancy between desired and real social relations leads to loneliness. Size of the network, frequency of contact with members and quality of the network are dimensions of an individual’s social network (31). Some of the traits of a lonely individual are being less trusting, more anxious and pessimistic, perceiving others around them more negatively and approaching social interactions in a defensive and hostile manner. However, a positive social relationship network provides a source of support, meaning and guidance in life (32). As age increases, the prevalence of loneliness also rises. A strong association between loneliness and depression has been found (31–33). A higher prevalence of loneliness is evident in women (50–65-year-old), single, separated, divorced or widowed, living in a rural setting, lower frequency of social interactions and smaller social networks (31).

## 3 Materials and methods

This study tried to determine the effects of social media addiction, problem solving skills, satisfaction with life and depression on academic self-efficacy, GPA and self-evaluated success of students as well as their predictors so that strategies can be developed to improve students’ productivity. A part of this study that analyzed social media addiction and its dimensions was presented in a conference by Polat and Aslan (34).

### 3.1 Study design and setting

Scales and parameters were determined based on researcher experiences on that field, literature and previous studies (1, 3, 28, 35). This study employed a descriptive and correlational design involving university students.

### 3.2 Participants and procedures

The survey was conducted hybrid both online and face to face to collect enough sample size. The questionnaire was created via Google Forms and also on paper. Approximately 50% of the survey instruments were collected online and the rest were collected in the paper form. The purposive sampling method was used with the selection criterion being university students. The online survey was sent to students by emails, WhatsApp, MS Teams, Instagram and other social media platforms. Students were assured about the anonymity and confidentiality of their participation in the survey and that they could refrain from participating in the survey whenever they wanted. Information about the study and the informed consent were included in the first part of the questionnaire, and participants could complete the following parts of the survey after consenting to participate. The primary sample size was based on a 20,000 population, calculated according to Cochran’s equation and 377 appropriate samples were finally confirmed with 5% margin of error and a 95% confidence level. Four hundred nineteen questionnaires were collected between October 2022 and December 2022 at Bingöl University. Therefore, a sufficient sample size was reached within the scope of this study.

### 3.3 Measures

Personal information, academic self-efficacy, social media addiction scale (adult form), problem solving skills scale (for adults), satisfaction with life scale, loneliness scale, short form and PHQ-9 depression scale based on our previous findings and publications were applied in this study.

#### 3.3.1 Personal information form

This form containing introductory information about the students participating in this study was prepared by the researchers. The questions posed to the students were related to age, gender, the class of study programme, resident place, social support, main source of support, years of using social media, frequency of using social media daily, time spent on social media daily and general grade average.

#### 3.3.2 Academic self-efficacy scale and academic success

ASES, self-evaluated- academic performance and GPA variables are used in that study to measure the academic performance of students related to other variables. GPA is a more objective method compared other two methods. It would be scientifically interesting to compare three academic performance measures with other variables.

This ASES scale was developed by Jerusalem and Schwarzer (36), while the Turkish adaptation of ASES was by Yılmaz et al. (37). This scale, which has a one-dimensional structure, consists of 7 items (I am always able to accomplish the tasks that need to be done during university education etc. items) related to the academic self-efficacy structure and graded based on a 4-point Likert scale. The lowest score that can be obtained from the scale is 7 and the highest score is 28. A high score indicates high self-efficacy. The scale showed good internal consistency with Cronbach’s alpha value of 0.696.

A single item, ‘*Satisfied with my academic performance compared to my classmates*’ was separately added for the student’s self-evaluation with 6-point Likert scale (1: strongly disagree, 2: disagree, 3: somewhat disagree, 4: somewhat agree, 5: agree, 6: strongly agree) with ranges (1–1,83 = strongly disagree; 1, 83–2,66 = disagree; 2,66-3,49 = somewhat disagree; 3,49-4,32 = somewhat agree; 4,32 -5,15 = agree; 5.15–6.0 = strongly agree). The current GPA of students was used to evaluate the academic success of students with other variables.

#### 3.3.3 Social media addiction scale - adult form

This scale was developed by Şahin and Yağcı (38), together with the Virtual tolerance (VT) and virtual communication (VC) sub-dimensions. The degree of participation in expressed in a 5 -point Likert scale (1: completely not suitable for me, 2: not suitable for me, 3: undecided, 4: suitable for me, 5: very suitable for me). The SMAC-AF consists of 20 items (I see social media as an escape from the real world, I stay on social media longer than I planned. Etc.) with 20–100 points. The Virtual tolerance (VT) sub-dimension consists of items 1 to 11, and Virtual communication (VC) consists of items 12 to 20. Items 5 and 11 were reverse scored. A high score means that the individual perceives himself as a “social media addict.” The level of addiction range developed by Şahin and Yağcı (38) is shown in Table 1. The Cronbach Alpha’s internal consistency coefficient for the overall scale was 0.94 and 0.92 for Virtual tolerance and 0.91 for Virtual communication (38). The Cronbach Alpha internal consistency coefficient for the overall scale in this study showed very good internal consistency with a Cronbach’s Alpha score of 0,876. Virtual tolerance and Virtual communication had very good Cronbach’s Alpha scores of 0,803 and 0,833, respectively.

**Table 1:** Social media addiction scale and evaluation of subscales (38).

Addiction level	SMAC-AF (1–20)	Virtual tolerance (1–11)	Virtual communication (12–20)
No addiction	20–36	11–19	9–15
Low dependency	37–52	20–28	16–22
Medium-intermediate dependency	53–68	29–37	23–30
Highly dependency	69–84	38–45	31–37
Very high dependency	85–100	46–55	38–45

#### 3.3.4 Problem solving skills for adults

This scale was developed by Yaman and Dede (39) and they carried out the validity and reliability of that scale in Türkiye. Problem solving skills scale for adults is a 5 -point Likert scale consisting of 18 items (I compare every possible solution to find the best solution to a problem etc.). Thinking about the effects of the solution to the problem (items 1–5), problem solving through modeling (items 6–8), alternative solutions research (items 9–12), determination in practice (items 13–15) and analyzing the problem encountered (16–18) are five sub–factors of the scale. The arithmetic mean scores ranges: Never (1–1.8), Seldom (1.81–2.60), Sometimes (2.61–3.49), Often (3.41–4.20) and Always (4.21–5.00) are used to compare with our results that higher scores show better problem solving skills for adults. The Cronbach’s Alpha internal consistency coefficient for the overall scale in this study was found to be ‘very good’ with a score of 0.934. The solution of the problem, problem solving through modeling, alternative solutions research, determination in practice and analyzing the problem encountered had a Cronbach’s Alpha score of 0,876, 0,752, 0,837, 0.768 and 0,784, respectively.

#### 3.3.5 Satisfaction with life

The adapted Turkish scale was developed by Lavallee et al. (40) and improved by Akın and Yalnız (41). The Life Satisfaction Scale consists of 5 items (I have a life close to my ideals in many ways, my living conditions are perfect etc.). This one-dimensional scale has a 7-point Likert rating (“1” absolutely disagree - “7” absolutely agree). The satisfaction in life scale was translated by Aslan, Ochnik & Çınar (2) and applied to university students in Türkiye. The possible range of scores is 5–35, with a score of 20 representing a neutral point on the scale. Cutoff scores are low (5–17), medium (18–23), satisfied (26–30), and high (24–35). In this study, the internal consistency coefficient for the satisfaction with life scale was very high with a Cronbach’s Alpha score of 0.872.

#### 3.3.6 UCLA loneliness scale short form

The UCLA Loneliness Scale Short Form (ULS-8) was developed by Hays and Dimatteo (42) and Dogan et al. (43) provided the Turkish adaptation. The scale consists 8 items (I do not have any friend, there’s no one I can turn to etc.) with a 4-point Likert-scale (1 - not suitable to 4 - completely appropriate) with two reversed items (items 3 and 6). The highest score that can be obtained from the scale is 32 and the lowest score is 8. As the scores obtained from the scale increase, the individual’s level of loneliness also increases (43). In this study, the internal consistency coefficient for the overall scale was found to be very high with a Cronbach’s Alpha score of 0.814.

#### 3.3.7 Depression scale

The PHQ-9 consists of 9 items (having little interest or pleasure in doing things, feeling sad, depressed or hopeless etc.) and it conforms with the DSM-V diagnostic criteria (44). Sari et al. (45) had adapted the reliability and validity of the PHQ-9 scale in Türkiye. The PHQ-9 is a measurement based on the patient health survey and there are 9 questions in the questionnaire and each question is scored on a 3-point Likert-scale ranging from 0 = not at all to 3 = nearly every day. Points are collected for each question and according to the scoring system in the original questionnaire, 1–4 points are rated as minimal, 5–9 light, 10–14 moderate, 15–19 moderate and 20–27 severe (depression). A cut-off score of 10 (PHQ-9 ≥ 10) or more is recommended for screening major depressive disorders (46, 47). In this study, the internal consistency coefficient for the overall scale was found to be very high with a Cronbach’s Alpha score of 0.870.

### 3.4 Statistical analysis

Descriptive statistics (reliability of the measures, frequencies, mean scores (M), standard deviation (SD) and percentages) were used in the preliminary statistical analysis. The normality assumption was verified using skewness and kurtosis scores of between −2 and + 2 as being acceptable (48). A one-way ANOVA and independent t-test were performed for testing the differences in mean scores. Next, correlation and Multiple linear regression methods were used to determine the correlation and predictors of the dependent variables.

Pearson correlation (>0.8) and Variance inflation factors (VIF) (< 5 indicates a low correlation, between 5 and 10 indicates a moderate correlation, > 10 indicates a high or intolerable correlation) of the model predictors (49) for collinearity diagnostics, while no pattern in the residuals (error) for homoscedasticity (equal variance, and no pattern) was determined by making a scatterplot with the residuals against the dependent variable. The Durbin Watson test (2 - no autocorrelation; 0 to <2 - positive autocorrelation and > 2 to 4 – negative autocorrelation) for no autocorrelation of the error terms (independent errors) and normally distributed errors by the quantile-quantile (Q-Q) plot or the probability-probability (P–P) plot were examined for linear regression assumptions in this study.

### 3.5 Ethical aspect of the research

This study was approved by the Bingöl University Health Sciences Scientific Research and Publication Board (Protocol N. E.74742), in accordance with the Declaration of Helsinki. The data collection form states that participation in this study is on a voluntary basis and the information provided by the participants would be treated with strict confidentiality. In addition, the purpose of the research and the eventual results were also explained. The participant’s confirmation to participate in the study (informed approval principle) was written in the data collection form.

## 4 Results of the study

### 4.1 Sample characteristics and descriptive statistics

The majority of participants were female (68,5%) and the average age was 21 years (21,35 ± 3,32) with an average income of 2.045 Turkish Lira (₺) per month. 46,1% of them were first class students and 67,5% of them received accommodation in a dormitory (See Table 2).

**Table 2:** Descriptive statistics and social media use of the study sample (*n* = 419).

Variable		*n*	%	Variable		*n*	%
Gender	Male	132	31,5	Place accommodated	Family	101	24,1
	Female	287	68,5		Dormitory	283	67,5
Class	Preparatory class	3	0,7		Friends	21	5,0
	1.class	193	46,1		Alone	14	3,3
	2. class	78	18,6	Social support	Yes	145	34,6
	3. class	79	18,9		No	274	65,4
	4. class	66	15,8	Daily visits to social media platforms	1–5 times	21,2	21,2
Using social media in years	Less than 1 year	59	14,1		6–10 times	30,3	30,3
	1–3 years	109	26,0		11–15 times	14,3	14,3
	4–6 years	138	32,9		More than15 times	34,1	34,1
	More than 7 years	113	27,0	Time spent daily social media platforms	Less than 1 h	46	11,0
Criticized by the family environment for using social media	Yes	146	34,8		1–3 h	195	46,5
	No	273	65,2		4–6 h	121	28,9
	Total	419	100,0		More than 7 h	57	13,6

Age average = 21,35 ± 3,32; Monthly income = 2.045,50; Grade average (GPA) = 71,17 ± 9,69(*n* = 218).

They received low levels of social support from friends and family members (65,4%). The average grade (GPA) was 71 (71 ± 9,69, *n* = 218) out of 100, indicating a moderate level of academic success. 32,9% had been using social media for 4–6 years and 27.0% had been using social media for more than 7 years and many started to use social media at an early age. 34,1% of them visited social media platforms more than 15 times per day and just 11,0% spent less than an hour on social media platforms, which indicates that 42,5% have the potential to become social media addicts. Moreover, 34.8% were criticized by family members for spending too much time on social media, as shown in Table 2.

Middle level (53,49 ± 14,59) social media addiction was measured among students. Sub-groups of the scale contained middle level virtual tolerance (30,99 ± 8,72) and low middle level virtual communication (22,49 ± 7,53) social media addiction, as shown in Table 3. The average academic self-efficacy total is 19,14 ± 3,89 with average mean:2,73 ± 0,55 indicating that they have high academic self-efficacy (2,5-3,25). ‘*Satisfied with my academic performance compared to my classmates*’ had a mean average: 4,06 ± 1,41 indicating that they somewhat agreed with their academic performance. The students had a mean of 3,75 ± 0,75 (sum 67,59 ± 13,65) for problem solving skills in the ‘*often range*’ (3,41-4,20). The sub-items were also in ‘*often range*’ and ‘*determination in the practice group*’ has the highest mean (3,92 ± 0,90). Students were slightly dissatisfied (18,47 ± 7,66) with their life and they had a mean of 15,29 ± 5,80 for loneliness, representing mild loneliness. They had a moderate depression level and 51,31% (PHQ > 10) of them had a type of depression. 8,6% of them had thought about death almost every day and 13,6% of them had thought of death for more than half a day.

**Table 3:** Descriptive statistics, prevalence and normality checking of the scales (*n* = 419).

Scale	Range	SumM ± SD	M ± SD	Skewness	Kurtosis
Social media addiction	20–100	53,49 ± 14,59	2,67±,72	,392	,103
Virtual tolerance	11–55	30,99 ± 8,72	2,81±,79	,320	–,150
Virtual communication	9–45	22,49 ± 7,53	2,49±,83	,434	–,036
Academic self-efficacy	7–28	19,14 ± 3,89	2,73±,55	–,215	,119
Problem solving skills for adults	18–90	67,59 ± 13,65	3,75±,75	–,453	,051
Thinking about the effects of the solution to the problem	5–25	18,69 ± 4,64	3,73±,92	–,722	,181
Problem solving through modeling	3–15	10,64 ± 2,66	3,54±,88	–,437	,224
Determination in practice	3–15	11,78 ± 2,70	3,92±,90	–,682	–,027
Analyzing the problem encountered	3–15	11,74 ± 2,78	3,91±,92	–,777	,150
Alternative solutions research	4–29	14,71 ± 3,58	3,67±,89	–,336	–,232
Satisfaction with life	5–35	18,47 ± 7,66	3,69 ± 1,53	,136	–,733
Loneliness	8–34	15,29 ± 5,80	1,91±,72	,475	–,651
Depression	0–27	10,47 ± 6,54	1,16±,72	,429	–,400

M, mean; SD, standard deviation; SumM, Average Sum; PHQ > 10:%51,31.

### 4.2 Checking the significant differences of the scales

According to the independent t-test on gender, significant differences were found for life satisfaction (*t* = 2,95; *p* = 0,003 < 0,05; Cohen’s d = 0,311) and depression (*t* = −2,27; *p* = 0,02 < 0,05; Cohen’s d = 0,23) scales, as shown in Table 4. Males had a higher life satisfaction (20,09 ± 7,71) with medium effect size, while females had higher depression levels with small effect size (Cohen’s d = 0,23). There were no significant differences in social support in all scales (*p* > 0,05). According to the one-way ANOVA test for ‘*staying place*’, there were significant differences for social media addiction (*F* = 2,981; *p* = 0,031 < 0,05; *η*^2^ (Eta squared) =0,021) with a 2,1% medium effect size, while there were no significant differences for other scales (*p* > 0,05). Significant differences were measured for ‘*staying with family*’ (51,33 ± 16,41) and ‘*friends*’ (61,57 ± 11,80), whereby students living with friends were more addicted to social media.

**Table 4:** Significant differences according to gender.

Scale	Gender	*N*	Sum Mean ± SD	*t*	*p*
Social media addiction	Male	132	54,77 ± 13,74	1,21	0,22
	Female	287	52,90 ± 14,94		
Academic self-efficacy scale	Male	132	19,58 ± 3,88	1,55	0,12
	Female	287	18,94 ± 3,88		
Problem solving skills	Male	132	67,07 ± 14,45	-0,52	0,60
	Female	287	67,82 ± 13,28		
Satisfaction with life	Male	132	20,09 ± 7,71	2,95	**0,003**
	Female	287	17,72 ± 7,53		
Loneliness	Male	132	15,75 ± 5,29	1,08	0,28
	Female	287	15,09 ± 6,02		
Depression	Male	132	9,40 ± 6,57	−2,27	**0,024**
	Female	287	10,96 ± 6,48		

Bold values indicate *p* < 0.05, significant differences.

There were significant differences for the social media addiction scale (*F* = 22,55; *p* = 0,000 < 0,05; *η*^2^ = 0,1,402), with a large effect size proportion of variance according to students’ daily usage determined by means of a one-way ANOVA test. The highest mean was measured for students using more than 7 h of social media (63,66 ± 17,16, *n* = 57) and between 4 and 6 h daily (57,47 ± 12,70, *n* = 121). Furthermore, there were significant differences in visiting social media platforms for the social media addiction scale (*F* = 29,59; *p* = 0,000 < 0,05; *η*^2^ = 0,176) and satisfaction with life (*F* = 4,25; *p* = 0,006 < 0,05; *η*^2^ = 0,029), indicating that students visiting social media more than 15 times per day (60,20 ± 15,07, *n* = 143) and 11–15 times per day (56,43 ± 10,37, *n* = 60) had the highest mean. Student groups who visited more than 15 times per day (17,73 ± 8,05, *n* = 143) and 11–15 times per day (17,48 ± 6,88, *n* = 60) had the lowest life satisfaction.

### 4.3 Correlation among variables

Social media addiction has the highest correlation with time spent on social media (*r* = 0,352; *p* < 0,01), loneliness (*r* = 0,341; *p* < 0,01) and depression (*r* = 0,307; *p* < 0,01), as well as a negative low correlation with academic self-efficacy, problem solving skills and life satisfaction, as shown in Table 5. Academic self-efficacy had the highest positive correlation with ‘*satisfied with my academic performance compared to my classmates*’ (*r* = 0,426; *p* < 0,01), problem solving skills (*r* = 0,411; *p* < 0,01), life satisfaction (*r* = 0,293; *p* < 0,01) and GPA (*r* = 0,253; *p* < 0,01), while a negative correlation with loneliness (*r* = −0,280; *p* < 0,01) and depression (*r* = −0,148; *p* < 0,01). Problem solving skills had the highest positive correlation with academic self-efficacy (*r* = 0,411; *p* < 0,01) and ‘*satisfied with my academic performance compared to my classmates*’ (*r* = 0,315; *p* < 0,01), while a negative correlation was measured with loneliness (*r* = −0,393; *p* < 0,01). Depression had the highest significant correlation with loneliness (*r* = 0,388; *p* < 0,01) and social media addiction (*r* = 0,307; *p* < 0,01), while a significant negative correlation with life satisfaction (*r* = −0,288, *p* < 0,01), academic self-efficacy (*r* = −0,148; *p* < 0,01) and monthly income (*r* = −0,145; *p* < 0,01).

**Table 5:** Correlation among variables.

Scales	1	2	3	4	5	6	7	8	9	10	11	12	13
1. Social media addiction	1	–,115^*^	–,154^**^	–,131^**^	,341^**^	,307^**^	–,077	–,164**	,416**	,140**	,352**	–,033	–,127*
2. Academic self-efficacy scale	–,115^*^	1	,411^**^	,293^**^	–,280^**^	–,148^**^	,116*	,018	–,015	,057	–,054	,253**	,426**
3.Problem solving skills	–,154^**^	,411^**^	1	,234^**^	–,393^**^	–,055	,117*	–,041	–,086	,113*	–,082	,161*	,315**
4. Satisfaction with Life	–,131^**^	,293^**^	,234^**^	1	–,248^**^	–,288^**^	,137**	,140**	–,133**	,085	-,081	,083	,235**
5. Loneliness	,341^**^	–,280^**^	–,393^**^	–,248^**^	1	,388^**^	–,076	–,059	,078	–,070	,060	–,033	–,268**
6. Depression	,307^**^	–,148^**^	–,055	–,288^**^	,388^**^	1	–,117*	–,145**	,021	,007	,079	,017	–,072

*Significant at the 0,05 level, **significant at the 0,01 level; 7 = age,8 = Monthly income,9 = Number of visits to social media pathforms,10 = Years of using social media,11 = Time spent on social media,12 = GPA, 13 = Satisfied with my academic performance compared to my classmates.

### 4.4 Multiple linear regression

A regression model that meets the sufficiency criteria based on logical connection of variables was developed to measure the academic success of students (academic self-efficacy, GPA and satisfied with academic performance), as the dependent variable, from differences in independent variables like gender (binary variable: 0 = male and 1 = female), age, yearly income, living place as four different binary variables vs. others (family = 1 vs. 0 = other etc.), class as binary variables vs. others (1.class = 1 vs. others = 0 etc.), years of internet usage, daily visits to social media platforms, daily time spent on social media and scales (social media addiction, loneliness, PHQ, problem solving, satisfaction with life) were entered into a multi linear regression model by using the stepwise method. Sub-scales of social media addiction and problem-solving scales in the models (Model 2, Model 4 and Model 6) were separately entered into the multi linear regression model instead of the main scale to measure their effects on students’ academic success.

The regression models provided in Table 6 shows a summary of significant predictive factors in terms of coefficients, B, for each variable obtained from the regression analysis and the best models are highlighted in the bold. Problem solving, satisfaction with life, 4.class & vs., loneliness and living alone were significant predictors of academic self-efficacy with an Adjusted R Square: 0,237 in Model 1 (Academic self-efficacy = 1,820 + 0,217*problem solving skills+0,066*satisfaction with life +0,219*4.class vs. others −0,103* loneliness +0,345* living alone vs. others). The model indicates that for every additional unit in problem solving skills, academic self-efficacy increases by an average of 0,217 if all other factors are kept constant. Similarly, other factors can be the models. The ‘*alternative solutions research*’ sub-group in the problem-solving skills scale was the only significant parameter, besides satisfaction with life, 4.class vs. others, loneliness and living alone vs. others, as predictors of academic self-efficacy with an Adjusted R Square: 0.09, as shown in Model 2 (Academic self-efficacy = 1,976 + 0,188*alternative solutions research+0,068*satisfaction with life+0,228*4.class vs. others −0,125* loneliness +0,356* living alone vs. others). 1.class vs. others, problem solving and gender in the third model of full scales with an Adjusted R Square: 0.075 in Model 3 (GPA = 61,310 + 2,175* problem solving skills+2,887* gender-8,513*1.class vs. others) and 1.class vs. others, ‘*thinking about the effects of the solution to the problem*’ and ‘*analyzing the problem encountered*’ by groups in the problem-solving skills scale with an Adjusted R Square: 0.24 in Model 4 (GPA = 65,61 + 3,52* thinking about the effects of the solution of the problem −1,87*analyzing the problem encountered −9,33*1.class vs. others), were the predictors of GPA. Problem solving, satisfaction with life and loneliness in Model 5 (Satisfied with academic performance = 2,41 + 0,435*problem solving skills +0,150*satisfaction with life-0,280*loneliness) with an Adjusted R Square = 0.145, and ‘*alternative solutions research*’, satisfaction with life and loneliness with an Adjusted R Square: 0.156 value in Model 6 (Satisfied with academic performance = 2,66 + 0,391*alternative solutions research+0,154*satisfaction with life-0,316*loneliness) were the predictors of satisfied with academic performance compared to other students.

**Table 6:** Regression model for academic success and self-efficacy of students.

Model 1 Academic self-efficacy	*B*	*t*	Sig,	Model 2 Academic self-efficacy	*B*	*t*	Sig,
Constant	1,820	10,186	0,000	Constant	1,976	13,134	0,000
Problem solving skills	0,217	6,185	0,000	Alternative solutions research	0,188	6,741	0,000
Satisfaction with life	0,066	4,105	0,000	Satisfaction with life	0,068	4,245	0,000
4.class vs. others	0,219	3,350	0,001	4.class vs. others	0,228	3,527	0,000
Loneliness	–0,103	–2,842	0,005	Loneliness	–0,125	–3,606	0,000
Living alone vs. others	0,345	2,629	0,009	Living alone vs. others	0,356	2,731	0,007
*F* Value	26,252		0,000	*F* value	8,07		0,000
Durbin-Watson	1,886			Durbin-Watson	,42		
Adjusted *R*^2^	0,237			Adjusted *R*^2^	0,091		
Δ*R*^2^ = 0,146(Model 1 better)							

Model 3 GPA	*B*	*t*	Sig,	Model 4 *GPA*	*B*	*t*	Sig,
Constant	61,310	15,753	0,000	Constant	65,61	18,930	0,000
1.class vs. others	–8,513	–2,749	0,007	1.class vs. others	−9,33	−3,068	0,002
Problem solving skills	2,175	2,252	0,025	Thinking about the effects of the solution of the problem	3,52	3,628	0,000
Gender	2,887	2,063	0,040	Analyzing the problem encountered	−1,87	−2,105	0,036
F Value	6,73		0,000	F Value	27,99		0,000
Durbin-Watson	0,38			Durbin-Watson	1,88		
Adjusted R^2^	0,075			Adjusted *R*^2^	0,249		
ΔR^2^ = 0,174(Model 4 better)							

Model 5 Satisfied with academic performance	*B*	*t*	Sig,	Model 6 *Satisfied with academic performance*	*B*	*t*	Sig,
Constant	2,410	4,799	0,000	Constant	2,660	6,264	0,000
Problem solving skills	,435	4,452	0,000	Alternative solutions research	0,391	5,017	0,000
Satisfaction with life	,150	3,316	0,001	Satisfaction with life	0,154	3,433	0,001
Loneliness	–,280	−2,761	0,006	Loneliness	−0,316	−3,250	0,001
F Value	23,05		0,000	*F* value	25,05		0,000
Durbin-Watson	1,90			Durbin-Watson	1,88		
Adjusted R^2^	0,145			Adjusted *R*^2^	0,156		

Δ*R*^2^ = 0,011 (Model 6 better).

Problem solving skills, mainly ‘*alternative solutions research*’, satisfaction with life, 4. class vs. others and living alone vs. others, are positive predictors of academic self-efficacy, while loneliness is a negative predictor of academic self-efficacy, as shown in Model 1 and Model 2. Problem solving skills and gender (being female) are positive predictors of GPA, while 1.class vs. others is a negative predicator of GPA, as shown in Model 3. ‘*Thinking about the effects of the solution to the problem*’ and ‘*analyzing the problem encountered*’ are both crucial problem-solving skills, while ‘*thinking about the effects of the solution to the problem*’ is a positive factor effecting GPA, while ‘*analyzing the problem encountered*’ is a negative predictor of GPA, as shown in Model 4. 1.class vs. others is again a negative factor of GPA. Problem solving skills, especially ‘*alternative solutions research*’ group and satisfaction with life, are positive factors affecting ‘*satisfied with academic performance compared with other students*’, while loneliness is a negative factor, as shown in Model 5 and Model 6 as shown in Table 6.

## 5 Discussion

Internet dependency, which leads to internet addiction, is rising among university students and has negative effects on their lives and academic performance. The 16 to 24 age group prefers to establish communications via social networks on the internet instead of face-to-face communication and establishing close relations with other individuals. This is a critical time in their social and emotional development that witnesses the highest internet usage, and thus, exposes them to more risks. University students are in the risk group due to problematic internet use in effort to seek acceptance and approval leading to risky decision-making that poses a threat to health and life in the form of excessive alcohol and cigarette use, drug abuse, driving recklessly, failure to fasten seatbelts etc. Family relations and academic life are also affected by increasing internet use. In addition, excessive social media use can cause emotional and identity problems (7). Medium to intermediate dependence on social media addiction was found in that study. Intermediate dependence in the virtual tolerance sub-scale and between low dependence and intermediate dependence in the virtual communication sub-scale was measured. Students living with friends were more addicted to social media. The social media addiction was found in students spending more than 7 h on social media (mean = 63,66) and visiting social media more than 15 times per day. Students with 15 visits per day and 11–15 visits per day, respectively, had the lowest satisfaction. The level of social media addiction should be decreased by spending less time and lowering visits to social media in order to improve productivity among university students.

Findings indicate that those who use social media for a longer time (4–7 h a day) are more addicted (50). 82,39% of students in Iran had various levels of addiction (51) and 95% of university students spend more than 4 h on social media in Malaysia (52). Some sources indicate that spending 4–5 h per day on the internet is considered an addiction. Most users spend a minimum of 3 h on social media, which could lead to some mental health problems. About 90% of university students have used social media for more than 4 years and 50,3% of them used it for more than 4 h daily at university. As students spend more time on social media and make more visits to social media networks daily, they become more addictive and join the risk group. Thus, it can be concluded that there is a moderate level of addiction among the University students (3, 4, 21). According to the results of the survey involving 114 students from Harran University in Türkiye, 36% of the students used the internet for 3 to5 h per day and 36% used the internet for 5 hours or more per day (53). This present study found that 46,5% of the university students spent between 1 and 3 h on social media, 28,9% of them spent between 4 and 6 h and 13,6% spent more than 7 h, while 42,5% of them have the potential of becoming addictive to social media, which is less than the previous years. Meanwhile, 34,1% of them visited the social media platform more than 15 times per day, 14,3% of them between 11 and 15 times and 30,3% of them between 6 and 10 times. About half of them visited social media platforms more than 10 times a day, indicating that they check their social media frequently (See Table 2).

Self-efficacy is an effective social behavior that strongly predicts students’ academic achievements and also acts as an important protective factor for psychological adjustment and health. It can also predict how people respond to an external threat or opportunity. Relevant responses toward problems are based on personal behaviors and efforts to achieve one’s objectives. Adopting positive problem-focused coping strategies by persons with high self-efficacy plays a critical role in stress relief (54). Students with high academic self-efficacy can evaluate their own academic performance compared to their classmates. The average grade obtained by 218 students in the above study was 71,17 or CC (Pass), based on Türkiye grade scoring. Overall, they possess middle level academic success and performance, even though they have high self-efficacy.

More than one-third of students experienced mental health problems even in the pre-pandemic period, where students were at a greater risk of depression than the general population (35). There was a 55,0% depression rate among university students in Türkiye during the second wave of COVID-19 (1), whereas, 36,8% of them had poor mental health compared to 25% with average health during the pre-pandemic era in the United Kingdom (55). Meanwhile, 74% of students were affected by depression in Bangladesh (56). The prevalence of depression was 40,3% during the first wave of the COVID-19 pandemic among university students in nine countries, with the highest prevalence in Türkiye (44,80%) and the lowest in Germany (4,80%). Several risk factors affected the declining mental health of students that female gender, younger age, and lower income were risk factors (35). In general, they had a moderate depression level and 51,3% of them (PHQ > 10) had some kind of depression in that study. Students from the lower income group were found to be affected by a volatile and worsening economic situation, high social media addiction and loneliness, which could the reason for this high prevalence. Conversely, students are still in the recovery stage and post-COVID-19 effects are the main reason for these mental health problems (See Table 3).

People can use the internet to overcome interpersonal relationship problems and cater for their intensive need of acceptance (57). Lack of social support can result in depression and anxiety symptoms and users try to prevent feelings of loneliness through social media by changing their social mood and avoiding problems in their daily life. Suicidal ideation is related to addiction behavior, depression, jealousy and emotional stress and is apparent among university students. The relationship between suicidal ideation and problematic use of the internet was measured. Cyber-victimization, online bullying, cyberbullying, explicating images without prior consent, name-calling, rumor mongering and harassment can lead to suicidal ideation (4). 8,6% of them thought about dying almost every day and 13.6% of them thought about it for more than half a day in that study. Low level social support from friends and family members (65,4%) can be another source of high levels of depression and suicidal ideation.

Some of the factors leading to higher internet addiction are lower age, gender (female), student, low education level, low income, low self-esteem, and narcissistic parameters (3). There were no differences in problematic internet use based on gender (23). Female students in Bangladesh showed 1,8 times more depression symptoms (56). It was also found that there was higher internet addiction among males but no gender differences in relation to problem-solving skills were found (16). The level of addiction was higher in younger Polish women (11). Aslan and Yaşar (3) applied the survey technique involving university students before the COVID-19 pandemic and found that male students spent more time on social media. It also found that males were more addicted compared to females (53). Bodur and Korkmaz (58) found that males used more social media, while Chung et al. (59) found that females spent more time on social media with a higher level of internet addiction. Problematic use of social media, such as being more unsocial or playing video games, was seen in males, while females were more addicted to social interaction (60). This present study found no significant differences in social media addiction based to gender. However, males had a higher mean of the internet addiction and females (43%) spent slightly more time (> 4 h) compared to males (41,2%). Furthermore, no significant differences were found for problem-solving skills, loneliness, and academic self-efficacy according to gender in the study. Differences were found in depression and satisfaction scales according to gender. Males were more satisfied (medium level of satisfaction) compared females and females were more depressed (See Table 4).

Loneliness refers to having limited social relationships. Higher scores on internet use indicate a higher degree of loneliness (57). Loneliness is correlated with internet addiction, and severe depressive symptoms were found among young Polish women (11). A positive relationship between students’ social media addiction levels and loneliness levels was found in Türkiye (61). There was a positive association between depression as well as emotional and social loneliness among university students (62). The academic performance of dependent students (lonelier) compared to other students on the internet was four times lower than non-dependent students’ performance (63). Negative correlation between social self-efficacy, loneliness and internet addiction, and a positive correlation between loneliness and internet addiction were found by Gazo et al. (64). Problem-solving training can be used as an intervention in parent–adolescent conflicts (17). Students from that study who used “*often to good*” (3,41 to 4,20) problem solving skills and the ‘determination during practice’ sub-group in that scale had the highest mean. They were slightly dissatisfied and experienced mild loneliness. Higher satisfaction, good problem-solving skills and social self-efficacy could counter depression and social media addiction.

Using social media for more than 5 h daily can lead to addiction and depression (12). Yearly or daily usage can have a positive correlation with social media addiction. Meanwhile, age was found to have a negative significant correlation with social media addiction (3). Using internet for research purposes leads to a higher proficiency in problem-solving skills related to their profession and lower internet addiction (16). There is a significant negative correlation between problematic internet use and academic self-efficacy (7). A moderate negative relationship between internet addiction, self-efficacy and problem-solving skills and a positive higher correlation between self-efficacy perceptions and problem-solving skills were found by İbili (16). The negative effect of internet addiction on adolescents’ later academic achievement was found to reduce academic achievement in China by reducing academic engagement and increasing dissatisfaction with learning activities (65). Internet addiction can cause psychological problems by decreasing academic achievement among adolescents (66, 67). There was a significant negative correlation between satisfaction with life and depression (1) and there was a significant negative correlation between academic self-efficacy and problematic internet use among university students in Karadeniz Technical University in Türkiye. Academic self-efficacy is a predictor of problematic internet usage (23). Social media addiction has the highest correlation with time spent on social media, loneliness and depression, while a significant negative correlation with academic self-efficacy, problem solving skills and life satisfaction that improved academic self-efficacy, problem solving skills and life satisfaction leading to better academic performance by decreasing the time spent on social media.

Students’ academic performance is negatively affected by social media addiction in the linear regression model (51). A stressful lifestyle can be alleviated by developing problem solving skills (17). Students’ GPA was significantly affected by social media addiction, whereby a higher level of addiction led to a decrease in the GPA as well as spending more time on social media instead of academic tasks and responsibilities (52). Problem solving (effects of solving and analyzing a problem), gender and classmates are predictors of GPA (dependent variable), since classmates is a negative predictor and gender (female) is a positive predictor. Problem solving (alternative solutions research), satisfaction with life and loneliness are predictors of satisfaction with academic performance, compared to classmates who are more satisfied with high problem-solving skills, show better academic success. Problem solving skills (alternative solutions research), satisfaction with life, classmates, and living alone are e significant positive predictors of academic self-efficacy, while loneliness is a significant negative predictor of academic self-efficacy among the University students.

Healthy internet use is important for students in efforts to achieve their objectives. Openness, loneliness and depression are three most important dimensions that describe social media addiction (4). Students (in Türkiye) facing social media addiction can be predicted based on their social anxiety and happiness levels through regression analysis (61). Self-control affects social media addiction by influencing GPA (68). Youth’s problem-solving skills can be developed with training programs (18). Interpersonal relationships, academic adjustment, as well as social and learning self-efficacies can be effective ways of handling smartphone addiction (54). Self-help and mindfulness, psycho-pharmacological therapy, cognitive-behavioral therapy, motivational interviewing and promoting clear use of policies and norms in schools and organizations through rewards and healthy use of social media are some of the methods used for overcoming social media addiction (4). Internet-based cognitive behavioral therapy intervention was found to be effective in dealing with students’ depression symptoms that affect academic performance (69). Conducting regular sporting activities can decrease internet addiction levels, while positively affecting problem-solving skills (16). An increase in physical activities by university students can lead to a decrease in mental health problems and internet addiction (70). Awareness among students regarding mental health services and the negative effects of social media on academic performance can be increased by healthcare providers and mental health services. Mental health literacy and stigma should be a part of the curriculum in universities (30, 70). Loneliness is a negative predictors on academic self-efficacy and satisfied with academic performance dependent variables based on our regression models that students are to be socialized and their Problem solving skills are to be improved. Analyzing the problem encountered, effects of the solution of the problem and alternative solutions research problem solving skills can be developed to improve the academic success of students and their resilience against mental problems. Ways to improve their satisfaction with life is also a positive predictor of both academic self-efficacy and satisfied with academic performance dependent variables. 1.class students need serious consultancy during their first year adaption and males are to encouraged to get better GPA.

### 5.1 Limitations of the study and directions for future researches

Current findings regarding social media addiction, loneliness, depression, life satisfaction, problem solving skills, and academic self-efficacy among university students will contribute to a detailed understanding of students’ academic success. There are some limitations to this present study, whereby results pertaining to the participant level were obtained via self-reported questionnaires relying on information obtained from students, which could lead to bias and thus, threaten the study’s validity. Participants comprised solely of students from Bingöl University, hence, curtailing the ability to generalize the results. The findings can help to develop better academic programs and cognitive-behavioral therapies, especially by psychologists, for students by developing social skills training with the aim of improving social relations among students.

This study can be repeated with other student populations. Other variables, such as problematic internet use, religiosity and personal traits, should also be investigated. Future research with a greater sampling scope and size is recommended to verify the results of this study. This study can be extended with different samples and different measures to determine and compare other addictive behaviors. Future studies can examine the interactive effects between stressful life events and diverse personal and psychological variables, whereby parent–child relationships, intimate relationships, and using cultural backgrounds with family demographic factors such as income, education level etc., can be measured.

This study highlights the vital function of engagement in learning activities and its relationship with internet addiction, depression etc., while adolescents’ academic achievements negatively influenced by internet addiction can be prevented by adopting protective factors and norms to reshape students’ behaviors. This would lead to the healthy use of social media and the coordination of families, governments and universities.

## 6 Conclusion

Moderate level social media addiction, especially higher virtual tolerance, mild loneliness and moderate depression levels among students was evaluated. Although they had high academic self-efficacy and problem-solving skills, they showed moderate GPA scores in reality and high levels of mental health problems. Students who visited social media platforms more than 15 times per day had the lowest satisfaction. Females were more depressed than males but males had higher social media addiction and loneliness. Students living with friends were more addictive based on the type of accommodation. Using more than 7 h of social media and visiting social media more than 15 times per day were the main factors leading to social media addiction. Students living with friends, female students, spending more time and visiting social media more frequently were risk factors for students’ academic success.

Students’ performance can be increased with higher levels of academic self-efficacy, problem solving skills and satisfaction with life. Problem solving skills, such as contemplating the effects of the solution on the problem and gender (female), are positive factors leading to higher GPA scores. Students with alternative solutions to research skills and higher satisfaction show better academic performance. Policymakers (governments, university management etc.) must initiate steps to decrease social media addiction and improve students’ performance by improving their problem-solving skills. Daily usage of social media can be decreased by banning or limiting social media usage in universities. Feelings of loneliness can be prevented if students can increase their social skills and participate in university activities. Group-style homework and training can be developed. Depressed students need supports from friends, family as well as psycho-social and consulting centers in their respective universities.

## Data availability statement

The original contributions presented in the study are included in the article/supplementary material, further inquiries can be directed to the corresponding author/s.

## Ethics statement

This study was approved by the Bingöl University Health Scientific Research and Publication Board for the research (protocol N. E.74742), in accordance with the Declaration of Helsinki. The studies were conducted in accordance with the local legislation and institutional requirements. The participants provided their consent to participate in this study.

## Author contributions

IA: Conceptualization, Data curation, Formal analysis, Funding acquisition, Investigation, Methodology, Project administration, Resources, Software, Supervision, Validation, Visualization, Writing – original draft, Writing – review & editing. HP: Conceptualization, Investigation, Methodology, Project administration, Resources, Validation, Writing – original draft, Writing – review & editing.
